# Characterization of the effect of pomegranate crude extract, and its post-harvesting preservation procedures, on redox tone, cellular growth and metabolic profile of MDA-MB-231 cell line

**DOI:** 10.1186/s12906-023-04134-1

**Published:** 2023-09-08

**Authors:** Aristide Ferrante, Mirko Tamma, Francesca Agriesti, Francesco Tucci, Piervito Lopriore, Maria Luisa Amodio, Giancarlo Colelli, Nazzareno Capitanio, Claudia Piccoli, Consiglia Pacelli

**Affiliations:** 1 Department of Clinical and Experimental Medicine, University of Foggia, 71122 Foggia, Italy; 2 Department of Agricultural Sciences, Food, Natural Resources and Engineering, University of Foggia, 71122 Foggia, Italy

**Keywords:** Pomegranate, Cancer, Mitochondria, Post-harvesting preservation procedures

## Abstract

**Background:**

Pomegranate is known for its beneficial properties due to its high content in antioxidants and might constitute a natural option for preventing and treatment of different pathologies including cancer. Since mitochondria are involved in tumorigenesis through ROS production and modulation of oxidative metabolism, we investigated the biological effects of pomegranate on cellular redox state, proliferation and metabolism in the breast cancer cell line MDA-MB-231 (MDA).

**Methods:**

MDA were treated for 24 h with graded concentration of filtered Pomegranate juice (PJ) and tested for metabolic Flux Analysis with XFe96 Extracellular Flux Analyzer, for proliferation using the xCELLigence System Real-Time Cell Analyzer and for intracellular ROS content by Confocal Microscopy Imaging.

**Results:**

Cells-treatment with freshly prepared pomegranate juice (PJ) resulted in a significant reduction of the intracellular ROS content already at the lower concentration of PJ tested. Additionally, it enhanced mitochondria respiration, and decreased glycolysis at high concentrations, inhibiting at the same time cell proliferation. As pomegranate is a seasonal fruit, assessment of optimum storage conditions preserving its bio-active properties was investigated. Our results indicated that storage conditions under controlled atmosphere for 30 days was able to enhance mitochondrial respiration at the same extent than freshly extracted PJ. Conversely, freezing procedure, though retaining the antioxidant and cell-growth inhibitory property, elicited an opposite effect on the metabolic profile as compared with fresh extract.

**Conclusion:**

Overall, the results of our study, on the one hand, confirms the preventive/therapeutic potential of PJ, as well as of its post-harvested processing, for cancer management. On the other hand, it highlights the intrinsic difficulties in attaining mechanistic insights when a multiplicity of effects is elicited by a crude mixture of bio-active compounds.

**Supplementary Information:**

The online version contains supplementary material available at 10.1186/s12906-023-04134-1.

## Introduction

Pomegranate (*Punica granatum* L.) has been used since ancient times in the traditional medicine of several cultures, particularly in the Middle East. Pomegranate fruit (arils), juice and peel extracts are rich sources of polyphenols such as tannins, anthocyanins, and flavonoids and hence possess potent antioxidant properties [1–3]. The antioxidant activity of polyphenolic compounds is due to their ability to scavenge free radicals, donate hydrogen atoms, and chelate metal ions. Specifically, the antioxidant capacity of pomegranate juice (PJ) is mainly attributed to the ellagitannin and punicalagin and it has been shown to be 3 times higher than that of red wine or green tea infusion [2]. In addition, PJ has favorable pharmacological properties and may protect against inflammation, cancer and neurodegeneration [4, 5]. In particular, the pomegranate constituents are shown to modulate transcription factors, pro- and anti-apoptotic proteins, cell cycle regulator molecules, protein kinases, cell adhesion molecules, pro-inflammatory mediators, and growth factors in various cancers such as skin, breast, prostate, colon, and lung cancers in cell culture systems, animal models and humans [6]. As regarding breast cancer cells, studies show that pomegranate might come out as a noticeable natural option for its treatment when the therapeutic strategies including surgery, chemotherapy, radiotherapy, and hormonal therapy cause serious side–effects for patients [7–9]. Recent discoveries pointed out that pomegranate reduces breast cancer aggressive phenotypic features such as invasion and motility [10], decreases inflammation and its impact on cancer progression [11], attenuates cell growth likely due to its antioxidant activity [12].

Mitochondria are believed to be a major intracellular source of reactive oxygen species (ROS) as a byproduct of the respiratory chain-mediated transfer of electrons to oxygen and its incomplete reduction. The respiratory chain complexes are themselves very sensitive targets for ROS with significant damaging effects [13]. Accordingly, it has been shown, that lowering ROS content results in an improvement of the oxidative phosphorylation (OxPhos) [14, 15]. Whether pomegranate affect mitochondrial oxidative function in breast cancer cells as consequence of its antioxidant activity has not been determined yet.

Given the beneficial properties as therapeutic potential of pomegranate, and considering that the fruits are highly susceptible to weight loss and decay during postharvest handling and storage [16, 17], determining optimum storage conditions for pomegranate fruit or its juice aiming at preserving its valuable nutritional components, is important for food industry. Several storage methods are available to extend the storage life and maintain fruit quality including thermal and non-thermal technologies [18]. For all the methods used, extensive studies reported the chemical and physical properties of the fruits, that are differently preserved, depending on the methods used [18–21]. However, nothing is reported about the ensued biological effects.

The overall aim of this study was to investigate the biological effect of PJ, on breast cancer cells reporting its effect on: (i) reducing oxidative stress, (ii) reducing proliferation rate of cancer cell, (iii) increasing mitochondrial function. Moreover, we determined the optimum storage conditions able to preserve PJ efficiency in modulating the mitochondrial oxidative metabolism.

## Materials and methods

### PJ extract

All PJ extracts, with the exception of thermal pasteurized PJ (TP-PJ) and high pressure processed PJ (HPP-PJ), were obtained from fresh pomegranate arils removed from fruits, variety ‘Wonderful’, grown in southern Italy were provided by “Masseria Fruttirossi srl”, from its orchards located in Castellaneta, Italy, in accordance with the relevan guidelines/regulations/legislation.

Pomegranate fruits were stored for 30 and 60 days in 50L-containers connected to a continuous humidified flow of (i) 3 kPa O_2_ + 10 kPa CO_2_ (Controlled Atmosphere), or (ii) air at both 4 and 8 °C.

Initially and at each sampling fruit were evaluated for quality and PJ was extracted homogenizing about 100 g of arils in an Ultraturrax (IKA, T18 Basic; Wimington, NC, USA) for 1 min; the homogenate was filtered through cheesecloth and immediately transferred for cell treatment. In some cases, arils from stored fruit were held in modified-atmosphere packaging for 5 days at 5 °C to simulate the ready-to-eat product before extracting the PJ. Frozen PJ was obtained holding fresh extracted PJ at -20 °C for 30 days before cell treatment. TP-PJ and HPP-PJ was obtained from commercially available fruit juices from Lome Superfruit (https://shop.lomesuperfruit.com/).

### Cell culture and treatment

MDA-MB-231 (MDA) cells, human caucasian breast adenocarcinoma were purchased from American Type Culture Collection (ATCC, Manassas, VA, USA). MDA is a highly aggressive, invasive and poorly differentiated triple-negative breast cancer cell line as it lacks oestrogen and progesterone receptor expression, as well as HER2 (human epidermal growth factor receptor 2) amplification [22]. The cells were cultured at 37 °C in a 5% CO_2_ humidified atmosphere in complete DMEM High Glucose medium (25 mM glucose), supplemented with 10% fetal bovine serum, penicillin–streptomycin (100 U/mL) and 2 mM L-glutamine. For PJ treatment, 0.5–2.0 × 106 cells/mL were supplemented with graded concentration of filtered PJ (2,5%, 5% or 10% v/v) for 24 h.

### Metabolic flux analysis

Oxygen consumption rate (OCR) and extracellular acidification rate (ECAR) were measured in adherent MDA cells with a XFe96 Extracellular Flux Analyzer (Seahorse Bioscience, Billerica, MA, USA) as described in [23]. 6000 cells/well were seeded in 96-well XF96 microplate 48 h before the measurements. For OCR analysis, the growth medium was replaced with 180 μL of bicarbonate-free DMEM supplemented with 25 mM glucose, 2 mM glutamine and 1 mM sodium pyruvate or with 2 mM glutamine and 1 mM pyruvate for ECAR measurements. Cells were preincubated at 37 °C for 45 min before starting the assay procedure.

OCR was measured under basal conditions and after sequential injection of oligomycin (1 μM), FCCP (0,5 μM), and rotenone + antimycin A (1 μM + 1 μM) to evaluate the ATP-linked respiration, the maximal respiratory capacity, and the non-mitochondrial oxygen consumption, respectively. For ECAR analysis, at the end of running, 50 mM 2-deoxyglucose was added in order to determine the glycolysis-independent acidification.

OCR and ECAR values were normalized to protein content in each well, determined using the BCA assay (Thermo Scientific, Waltham, MA, USA).

### xCELLigence assay

The xCELLigence System Real-Time Cell Analyzer (ACEA Biosciences, San Diego, CA, USA) was used for monitoring proliferation of cancer cells. MDA cells were seeded at a cell density of 6000 cells/well in 16-well E-plates, connected to the RTCA platform placed into a cell incubator at 37 °C and 5% CO2. When cells reached log growth phase, approximately 24 h after seeding, medium was removed, and cells were treated for 24 h with pomegranate juice or PBS as vehicle. Cell proliferation was monitored every 30 min for a period of up to 96 h, measuring changes of electrical impedance as a dimensionless parameter termed cell index (CI). CI was normalized just before treatment and converted into normalized cell index (NCI), by the RTCA-integrated software (Version 2.0, ACEA Biosciences, San Diego, CA, USA). Normalized cell index was calculated as described in [24] as follows: NCI = CI end of treatment/CI normalization time.

### Confocal microscopy imaging

Confocal microscopy Imaging was performed as described in [24]. Briefly, cells were cytospun on fibronectin-coated (Cell-tak-coated (Corning, New York, USA)) 35-mm glass-bottom dishes (Ibidi, Gräfelfing, Germany)] and incubated for 20 min at 37 °C with 5 μM DCF-DA (2,7-dichlorofluorescin diacetate) (Molecular Probes, Eugene, Oregon, USA) to detect cellular peroxide. Stained cells were washed with PBS and examined by a Leica (Wetzlar, Germany) TCS SP8 confocal laser scanning microscopy system utilizing appropriate excitation laser beams (images collected using a 60X objective (1.4 NA). Acquisition, storage, and analysis of data were performed with LasX software from Leica or ImageJ 1.48 (Wayne Rasband, NIH, Bethesda, Maryland, USA).

### Statistical analysis

Data are reported as mean ± standard error mean (SEM) of at least three independent experiments and compared by one-way or two-way ANOVA, followed by Bonferroni post-hoc test; a *p-value < 0.05 was accepted as statistically significant. All analyses were performed using GraphPad Prism Software Version 8 (GraphPad Software Inc., San Diego, CA, USA).

## Results

### Effect of pomegranate juice (PJ) on ROS content and proliferation in MDA cells

**Fig. 1:**
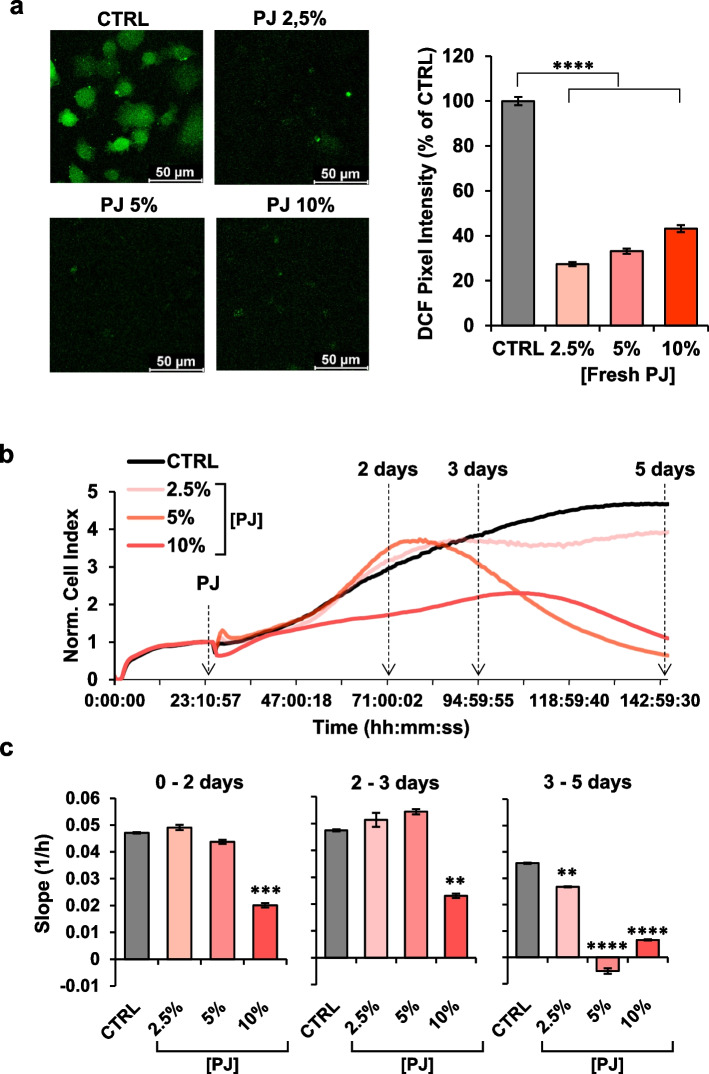
Effect of PJ on ROS content and proliferation in MDA cells. **a** Laser scanning confocal microscopy (LSCM) imaging of MDA treated with the redox probe dichlorodihydrofluorescein (DCF). Left panels show representative images of MDA cells treated with different PJ concentration. The histogram on the right shows statistical analysis of the DCF pixel intensity per cell normalized to control. **b** Representative Normalized Cell Index (NCI) real-time kinetics of cells growth exposed to increase of PJ concentration assessed by xCELLigence System Cell Analyzer. Cells were grown in complete DMEM medium for approximately 24 h after seeding, then medium was changed, and cells were treated for 5 days with DMEM medium containing different concentrations of PJ. Each cell index value was normalized just before this starting point. **c** The histogram displays the slope values calculated between the indicated time-points and at different PJ-concentration. All data are presented as mean ± SEM of two independent experiments carried out in triplicate under each condition. ** *p* < 0.01; *** *p* < 0.001; **** *p* < 0.0001. Statistical significances are referred to the controls

The antioxidant effect of PJ is widely reported in literature both in vitro and in vivo studies [25]. To verify the antioxidant property of freshly extracted PJ in our experimental model, we determined intracellular ROS content by the fluorescence probe DCF at different PJ concentrations in MDA cells, an epithelial human breast cancer cell line. As shown in Fig. 1a, PJ-treatments of cells for 24 h resulted in a significant reduction of the basal DCF-related signal already at the lower concentration of PJ tested (i.e. 2.5% v/v). These results confirmed the ROS scavenging activities of PJ constituents in our experimental conditions in agreement with previously reported evidence [15, 26]. Next we tested the impact of fresh PJ on MDA cell line proliferation using real-time monitoring of cell growth by the xCELLigence System Cell Analyzer for 5 days. As shown in Fig. 1b, MDA cell growth was significantly delayed at 10% of PJ concentration already after 48 h of treatment, whereas 5% and 2.5% PJ had effect only after 5 days of incubation. The cell index, that is a parameter reflecting the biolgical statue of monitored cells, including the cell number, cell viability, morphology and adhesion degree [27], were calculated at different time point (2, 3 and 5 days) in order to monitor the differences between different PJ treatments over the time. The results confirmed the dramatics effect of 10% PJ at each time point tested (Fig. 1c). These results would suggest that the antioxidant and cell growth-inhibiting effects of fresh PJ are apparently not directly correlated.

To rule out that the above observed effects were partly due to pH changes of the culturing medium following supplementation of the PJ we carried out control experiments with the pH of the medium buffered at the lowest pH attained with 10% v/v PJ. No significant effects were observed neither on the ROS scavenging activity or the cell growth (Fig S6). Similar controls were carried out for the further assays reported ahead always resulting in negative outcomes (Fig S6).

### Effect of PJ on mitochondrial function in MDA cells

**Fig. 2:**
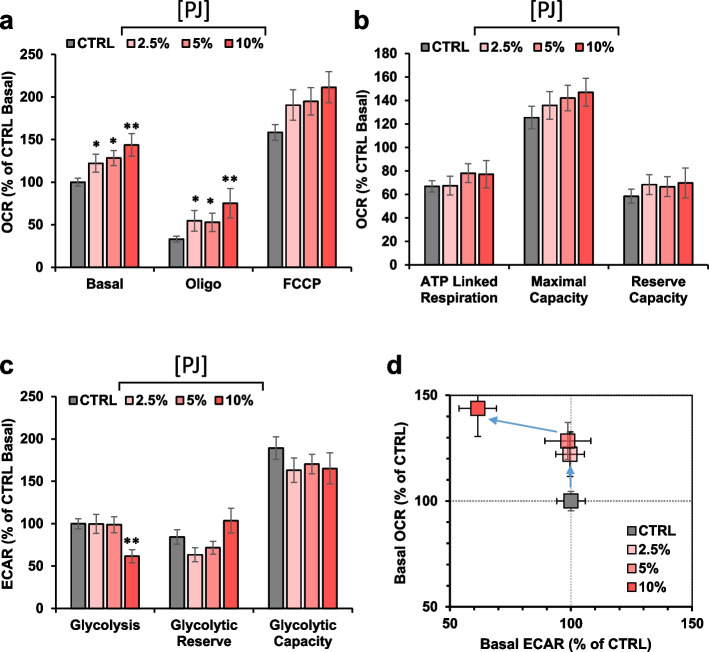
Comparative metabolic flux analysis of MDA cells treated with fresh PJ. Cells were analyzed in microplates by Seahorse XF 96 according to the Mito Stress protocol as described in material and methods. **a** Statistical analysis of mitochondrial respiration stress test assay. The histograms show Basal, resting OCR; oligo, OCR in the presence of oligomycin; FCCP, OCR under uncoupled condition. Data, means ± SEM are expressed as percentage of control Basal OCR (0.56 ± 0.06 pmol O_2_/min /µg protein). **b** The histograms show ATP turnover (i.e., basal – oligomycin), maximal capacity (OCR in the presence of FCCP) and reserve capacity (i.e., “max” – basal). Data, means ± SEM are expressed as percentage of control Basal OCR (**c**) Statistical analysis of glycolytic activity stress assay. The histograms show basal glycolysis, glycolytic reserve (i.e., oligo – basal ECAR) and glycolytic capacity (i.e. oligo- 2Deoxy-glucose). Data, means ± SEM are expressed as percentage of control Basal ECAR (0.493 ± 0.05 mpH/min /μg protein) (**d**) Energy map was obtained plotting basal OCR versus glycolysis ECAR. * *p* < 0.05. Statistical significances are referred to control. Abbreviations: oligo, oligomycin; FCCP, carbonyl cyanide 4-(trifluoromethoxy)phenylhydrazone; OCR, oxygen consumption rate; ECAR, extracellular acidification rate

As mitochondrial respiration plays an important role in producing energy by OxPhos and it is also a major source of cellular ROS production, we tested on it the effect of fresh PJ treatment. To this aim we performed a systematic analysis using the Seahorse technology to assess the two major cellular metabolic fluxes, the mitochondrial respiratory activities driving the OxPhos, measured as oxygen consumption rate (OCR), and glycolysis, measured as extracellular acidification rate (ECAR), mainly due to conversion of pyruvate to lactate, as previously described [23]. Using this methodology, the effect of fresh PJ at three different concentrations was tested on the metabolic fluxes in MDA cells. As shown in Fig. 2a, PJ treatment resulted in a dose-dependent increase of the OCR under conditions measuring i) basal, ii) ATP-synthesis inhibited and iii) maximal activities. Further analyses on the three parameters resulted in a PJ-mediated tendency to an increase in the ATP-linked respiration, maximal respiratory capacity and reserve capacity (Fig. 2b) which, however, reached statistical significance at the highest concentration of PJ tested as compared with the untreated cells. Unlike OCR, ECAR (i.e. glycolysis) showed not significant change, with a tendency to decrease though non statistically significant at 10% of PJ treatment (Fig. 2c). A similar trend but in the opposite direction was observed for the glycolytic reserve whereas no changes where observed for the glycolytic capacity at all the PJ concentrations tested. Correlation of the basal OCRs and ECARs in the energy map (Fig. 2d) clearly showed that PJ induced a metabolic shift in MDA cells toward a more energetic/more oxidative metabolic phenotype.

### Effect of pomegranate juice (PJ) processed with thermal pasteurization (TP) and High Pressure Processing (HPP) on mitochondrial function in MDA cells

**Fig. 3:**
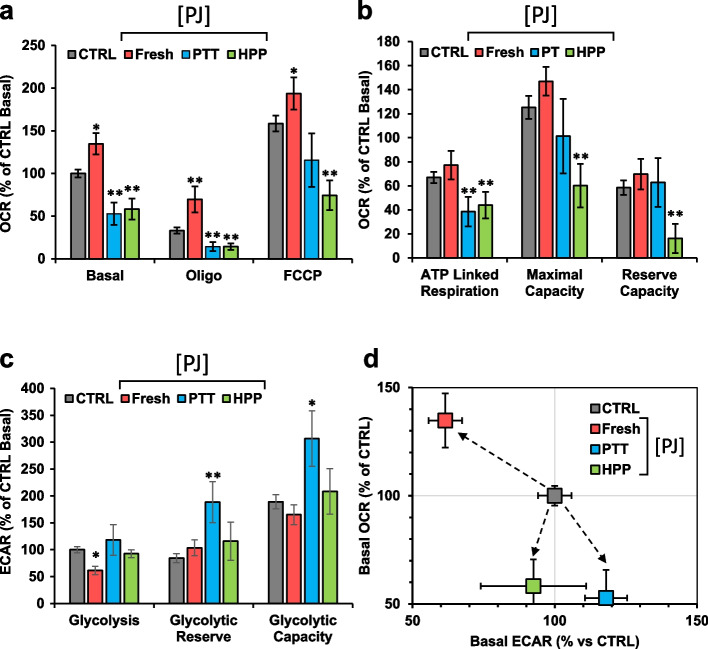
Effect of PJ processed with thermal pasteurization (PT) and high hydrostatic pressure (HHP) on mitochondrial function in MDA cells. MDA were incubated 24 h with fresh, thermal pasteurization (PT) or high pressure pasteurization (HPP) PJ and analyzed in microplates by Seahorse. **a** Statistical analysis of the OCRs obtained from Mitostress assay. The histograms show Basal, resting OCR; oligo, OCR in the presence of oligomycin; FCCP, OCR under uncoupled condition. Data, means ± SEM are expressed as percentage of control Basal OCR. **b** The histograms show ATP turnover, maximal capacity and reserve capacity. Data, means ± SEM are expressed as percentage of control Basal OCR (**c**) Statistical analysis of glycolytic activity stress assay. The histograms show basal glycolysis, glycolytic reserve and glycolytic capacity. Data, means ± SEM are expressed as percentage of control Basal ECAR. **d** Energy map was obtained plotting basal OCR versus glycolysis ECAR; * *p* < 0.05, ** *p* < 0.01; *** *p* < 0.001. Statistical significances are referred to control

Freshly made PJ can be immediately used for fresh market, but its quality lasts only a few days in a cold storage. Therefore, alternative methods of processing and storage are needed which can increase microbiological stability and preserve nutritional and bioactive properties [28]. Thermal pasteurization (TP) and High Pressure Processing (HPP) are technologies currently used for preservation and extension of the shelf-life of juices [29]. To evaluate if these treatments preserved the metabolic properties observed for fresh PJ we assessed OCR and ECAR in the MDA cells incubated with 10% TP-treated PJ (TP-PJ) and HPP-treated PJ (HPP-PJ). As shown in Fig. 3a, irrespective of the TP or HPP processing, treated PJ caused a significant decrease of the OCR both in all the conditions tested (i.e. basal, + oligo, + FCCP), as well as in the other derived bioenergetics parameters (i.e. ATP-linked respiration, maximal respiratory capacity and reserve capacity; Fig. 3b). Some difference was observed between TP-PJ and HPP-PJ with the latter being more effective in inhibiting maximal activity and capacity and reserve capacity. No large compensatory effects were observed on ECAR (Fig. 3c). Correlation of the basal OCRs and ECARs in the energy map clearly showed that both TP-PJ and HPP-PJ inverted the metabolic shift observed with fresh PJ inducing a quiescent/glycolytic phenotype in MDA cells (Fig. 3d).

### Effect of pomegranate juice (PJ) from pomegranate stored in Controlled atmosphere (CA) and in Regular air (RA) on mitochondrial function in MDA cells

**Fig. 4:**
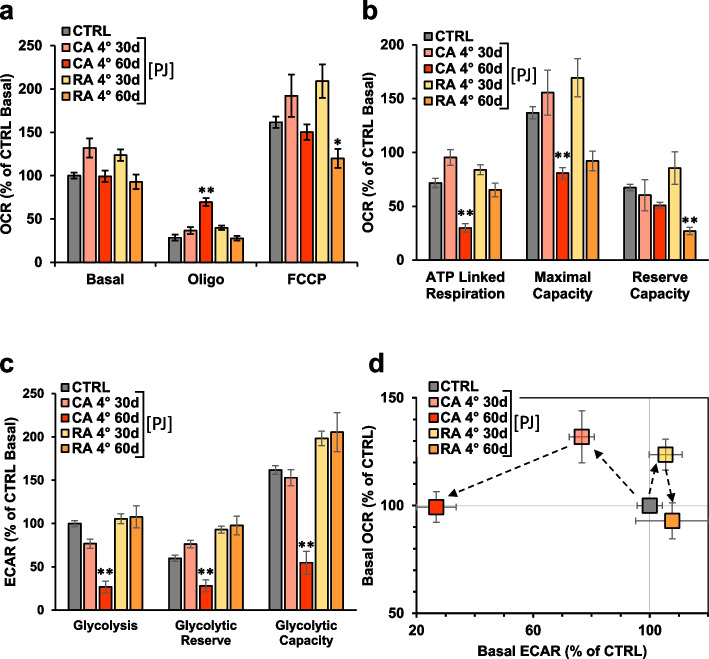
Effect of PJ from pomegranate stores in Controlled atmosphere (CA) and in regular air (RA) on mitochondrial function in MDA cells. Cells were incubated 24 h with PJ obtained from CA or RA stored pomegranate fruit for 30 or 60 days and and analyzed in microplates by Seahorse. **a** Statistical analysis of the OCRs obtained from Mitostress assay. The histograms show Basal, resting OCR; oligo, OCR in the presence of oligomycin; FCCP, OCR under uncoupled condition. Data, means ± SEM are expressed as percentage of control Basal OCR. **b** The histograms show ATP turnover, maximal capacity and reserve capacity. Data, means ± SEM are expressed as percentage of control Basal OCR. **c** Statistical analysis of glycolytic activity stress assay. The histograms show basal glycolysis, glycolytic reserve and glycolytic capacity. Data, means ± SEM are expressed as percentage of control Basal ECAR. **d** Energy map was obtained plotting basal OCR versus glycolysis ECAR; * *p* < 0.05, ** *p* < 0.01; *** *p* < 0.001. Statistical significances are referred to control

It has been reported that controlled atmosphere (CA) storage is a better option for pomegranate fruit storage than regular air (RA) in prolonging the shelf life of fresh products [30]. In order to evaluate if these different methods for pomegranate storage affect the mitochondrial respiratory function, we treated the MDA cells with PJ extracted from either CA-stored (CA-PJ) and RA-stored (RA-PJ) pomegranate whole fruit (30 and 60 days’ storage at 4 °C). As shown in the Fig. 4a, 10% of PJ treatment confirmed the increase of mitochondrial respiration, both under basal and maximal conditions when pomegranate fruits were stored for no longer than 30 days irrespective whether under CA or RA regimen. The observed PJ-mediated enhancement of the respiratory activity was lost if storage was prolonged up to 60 days. Unlike OCR, glycolysis decreased a little only with CA-PJ after 30 days’ storage and more substantially after 60 days’ storage. Correlation of the basal OCRs and ECARs in the energy map clearly showed that only the 30 days-stored CA-PJ caused a switch toward an oxidative phenotype (Fig. 4d). Interestingly, when we evaluated the CA-PJ stored at 8 degrees (Supplemental Fig. 1a-d), we did not find differences when compared with the results attained at 4 °C (Fig. 4). Additionally, when we analyzed the mitochondrial respiration and glycolysis in cells treated with PJ extract from arils packaged for 5 days after 30 days of whole fruits storage in CA or RA, no significant effect was found (Fig. S2). In particular, the pro-oxidative metabolic switch observed with the CA storage was lost. All together these results confirm that only the CA storage at 4 and 8 degrees for no longer than 30 days is able to substantially preserve the bioenergizing activity of pomegranate fruit.

### Effect of PJ-frozen storage of on mitochondrial function in MDA cells

**Fig. 5:**
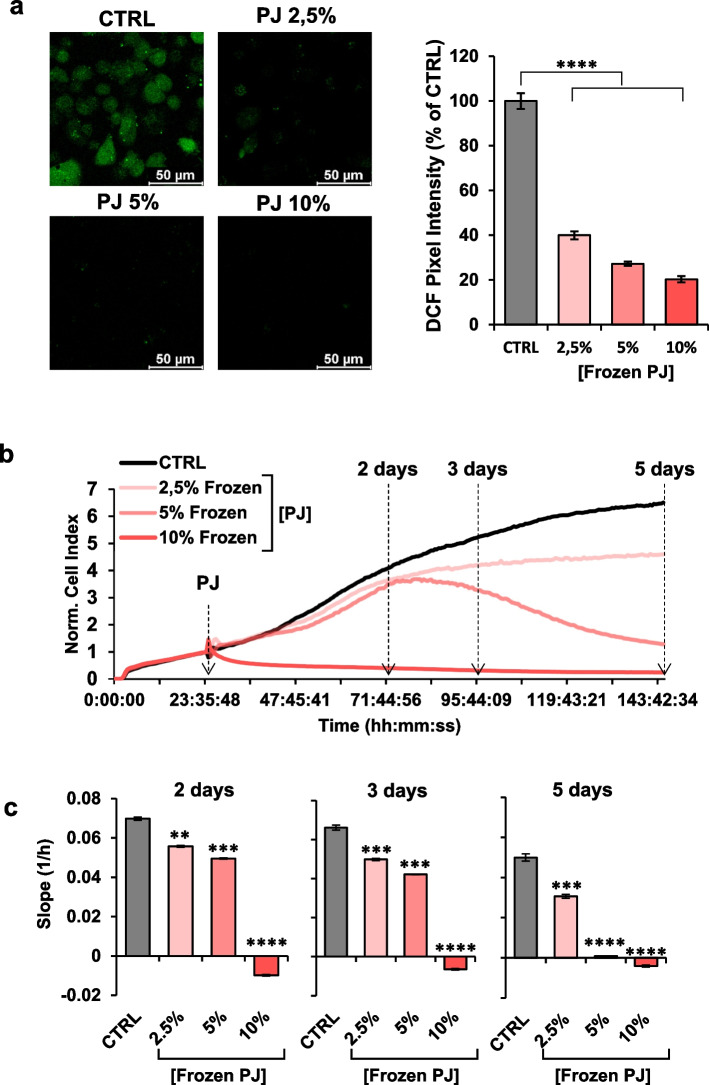
Effect of PJ-frozen storage on mitochondrial function in MDA cells. PJ was stored at -20 °C for 30 days, then MDA cells were incubated 24 h with DMEM medium containing different concentrations of frozen PJ. **a** Laser scanning confocal microscopy (LSCM) imaging of MDA treated with the reactive oxidant species probe DCF. Left panels show representative images of MDA cells treated with different PJ concentration. The histogram on the right shows statistical analysis of the DCF pixel intensity per cell normalized to control. **b** Representative Normalized Cell Index (NCI) kinetics of cells exposed to increase of PJ concentration. Cells were grown in complete DMEM medium for approximately 24 h after seeding, then medium was changed, and cells were treated for 5 days with DMEM medium containing different concentrations of PJ. Each cell index value was normalized just before this starting point. **c** The histogram displays NCI values at different days and different PJ-concentration of MDA cells. All data are presented as mean ± SEM of two independent experiments. ** *p* < 0.01; *** *p* < 0.001; **** *p* < 0.0001. Statistical significances are referred to the controls

**Fig. 6:**
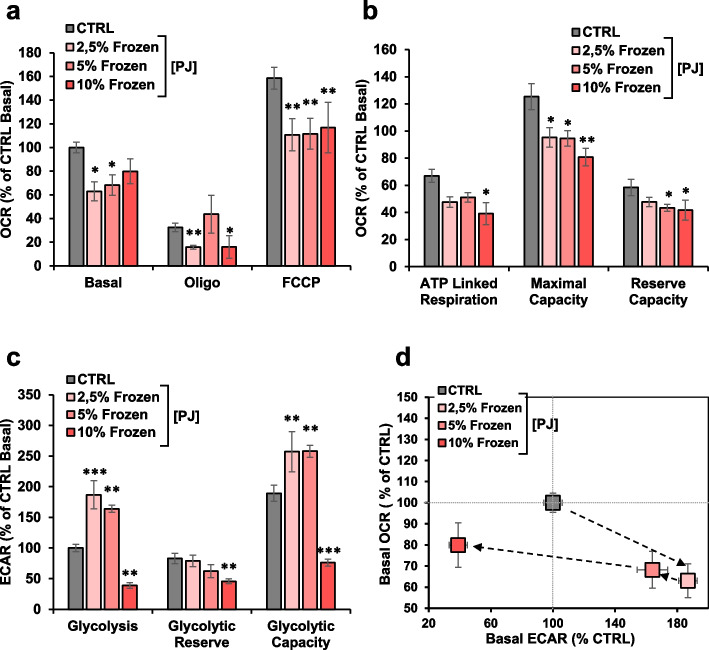
Comparative metabolic flux analysis of MDA cells treated with frozen PJ. Cells were analyzed in microplates by Seahorse XP 96 according to the Mito Stress protocol as described in material and methods. **a** Statistical analysis of mitochondrial respiration stress test assay. The histograms show Basal, resting OCR; oligo, OCR in the presence of oligomycin; FCCP, OCR under uncoupled condition. Data, means ± SEM are expressed as percentage of control Basal OCR (0.56 ± 0.06 pmol O_2_/min /µg protein). **b** The histograms show ATP turnover (i.e., basal – oligomycin), maximal capacity (OCR in the presence of FCCP) and reserve capacity (i.e., “max” – basal). Data, means ± SEM are expressed as percentage of control Basal OCR (**c**) Statistical analysis of glycolytic activity stress assay. The histograms show basal glycolysis, glycolytic reserve (i.e., oligo – basal ECAR) and glycolytic capacity (i.e. oligo- 2Deoxy-glucose). Data, means ± SEM are expressed as percentage of control Basal ECAR (0.493 ± 0.05 mpH/min /μg protein) (**d**) Energy map was obtained plotting basal OCR versus glycolysis ECAR. Statistical significances are referred to control: * *p* < 0.05; ** *p* < 0.01; *** *p* < 0.001

Freezing is one of the common procedure to preserve food materials such as fruit juices, but it is unknown whether the juice’s nutritional value is preserved during prolonged frozen storage. To address this issue, we analyzed ROS content and proliferation in MDA cells treated with frozen PJ (FZ-PJ) prepared from fresh pomegranate. Figure 5a shows that the antioxidant activity of FZ-PJ was comparable with that observed for fresh PJ being effective even at 2.5% concentration. Monitoring MDA real time growth by the xCELLigence System, revealed that, differently from what observed with fresh PJ, it was significantly delayed after 48 h and 72 h treatment even at concentrations of 2.5% and 5% (Fig. 5b). At 10% of FZ-PJ and after 5 days’ treatment the cell growth was dramatically inhibited as for the fresh PJ (Fig. 5c). Finally, assessment of the metabolic fluxes by Seahorse revealed that the switch toward a more oxidative metabolism, observed following treatment with fresh PJ, was completely abrogated in cells treated with FZ-PJ (Fig. 6a-d). Combination of the freezing procedure with either PT and HPP pre-treatment as well as with CA or RA storage was never able to recapitulate the pro-energetic/pro-oxidative metabolic phenotype observed with freshly extracted PJ (Supplemental Figs. 3a-d, 4a-d, 5a-d).

## Discussion

Natural products have been used for the treatment of various diseases and are becoming an important research area for drug discovery in cancer. These products have been extensively studied and have exhibited anti-carcinogenic activities by interfering with the initiation, development and progression of cancer through the modulation of various mechanisms including cellular proliferation, differentiation, apoptosis, angiogenesis, and metastasis. This concept is gaining attention because it is a cost-effective alternative to cancer treatment. Accumulating scientific evidences show that daily consumption of a diet rich in fruits and vegetables reduces the risk of cancer [31] and growing interest is focusing on plant foods containing polyphenolic compounds [32] that show tremendous anticancer properties targeting multiple signaling pathways [33].

In this context, pomegranate fruits is gaining high considerations for its strong antioxidant and anti-inflammatory properties due to their high content of hydrolysable tannins and anthocyanins [34] that have a broader range of action against several types of free radicals [35]. Consistent with this notion we confirmed the powerful antioxidant property of pomegranate juice extract in scavenging pro-oxidant reactive species even at low concentration in MDA-MB-231 cells, as reported in previous studies [36, 37]. The fluorescent probe DCF that we used in this study is widely utilized to assess reactive oxygen species although it has been reported that DCF lacks of specificity [38] being reactive also toward nitrogen reactive species and oxidized sulfhydryl moieties. Therefore, we cannot conclude which specific reactive species are effectively scavenged neither their intracellular localization.

However, the commonly accepted correlation between maintenance of a pro-oxidative tone and control of cell growth and proliferation is over-simplified and requires deepening as negative outcomes on cancer cell growth have been also reported. Indeed, several evidence suggests that ROS, or more generally a pro-oxidative state, within cells act as second messengers in intracellular signaling cascades which induce and maintain the oncogenic phenotype of cancer cells, due to their ability to increase cell proliferation, survival, cellular migration participating in the Ras-Raf-MEK1/2-ERK1/2 oncogenic signaling, and also by inducing DNA damage leading to genetic lesions that initiate tumorigenicity and sustain subsequent tumor progression [39]. In this context, several studies have documented the benefits of antioxidant drugs for cancer therapies [40, 41]. However, the use of antioxidants remains a clinical concern and ROS can behave as a double-edged sword since recent studies have shown that some classical antioxidants are known to cause toxicity on their own. It has been reported an increase in tumor development and metastasis in mouse models treated with vitamin E [42] as well as it has been shown that the administration of antioxidants, such as N-acetylcysteine, accelerates the progression of lung cancers and melanomas [43, 44].

Indeed, our results show that relatively low concentrations of PJ, that effectively remove basal reactive species content, have practically no effect on the growth of MDA cancer cell line after 48–72 h post treatment. Significant inhibition of cell growth is observed at higher concentration of PJ or after much longer treatment. Given the multiplicity of bioactive compounds present in the PJ it is possible that some of those, present at high concentration, are responsible for the antioxidant effect whereas other, present at lower concentration, are responsible for the slowdown/arrest of cell growth. Anthocyanidins, ellagic acid, ursolic acid, luteolin, punicalagin among the others are all PJ constituent proved to inhibit cell proliferation when used as pure compound. Although it would be informative to assess the compositional profile of the major bioactive compounds present in the PJ extract this was not the scope of the present study.

Mitochondria are a major intracellular source of ROS as a byproduct of respiration. Leak of electrons from the respiratory chain complexes directly to di-oxygen causes its incomplete reduction to the superoxide anion radical, that is the germinal species generating further reactive oxygen as well as nitrogen species. Because of the proximity to the ROS generating sites the respiratory complexes are themselves potential targets of oxidative modification [8]. This would establish a vicious cycle with deleterious impact on the cell viability if the ROS production exceeds the endogenous anti-oxidant defense. This notion is consistent with a large number of evidences showing that lowering ROS content results in improvement of the OxPhos. [14, 15]. Accordingly, the results shown in this study confirm previous observations. Indeed, concentrations of PJ that reduce the intracellular ROS content, cause at the same time a progressive increase of the basal mitochondrial respiratory activity (OCR was almost doubled at 10% PJ). Conversely, the glycolysis flux was unaffected at low and intermediate PJ concentrations and even reduced at the highest PJ concentration tested. As a consequence, the metabolic profile of MDA undergo a shift toward a more oxidative phenotype.

Although the link herein shown between the observed PJ-mediated pro-oxidative metabolic shift and the dampening of the cell proliferation is at the correlative level nevertheless it is insightful. Most cancer cells are known to rely on aerobic glycolysis even when oxygen availability is not limiting (Warburg effect) [27]. Aerobic glycolysis provides to proliferating cells energy and biosynthetic precursors [28]. However, this old view of cancer bioenergetics, has been challenged by an increasing body of evidence demonstrating that the bioenergetic phenotype of a given tumor can vary widely from glycolytic to oxidative phosphorylating, depending on the oncogenes activated, the developmental stage, the microenvironment [45]. Metabolic therapies to treat cancer are emerging thanks to the development of drugs able to inhibit or to enhance specifically a given metabolic pathway [46]. The efficacy of these therapies, however, strongly depends on the fine characterization of the onco-metabolic profile of the tumor to be targeted.

In line with this notion our group has provided in vitro evidence that utilization of inhibitors of metabolic pathways differentially affects the viability of a given cancer cell line depending on their specific metabolism [24, 47]. Relevant in this contest is our reported observation that enhancing pyruvate oxidation by DCA, an activator of the pyruvate dehydrogenase complex, inhibited proliferation of cancer cells the more they relied on aerobic glycolysis. Hormone independent breast cancer cell line MDA is one of the most studied estrogen receptor (ER) negative breast cancer cell lines. This cell line is known to be highly metastatic, invasive, and glycolytic [48]. Treatment of MDA-MB-231 cells with freshly prepared PJ enhanced mitochondria respiration, and decreased glycolysis at high concentration, inhibiting at the same time cell proliferation.

The above described results were obtained with PJ extract from pomegranate immediately harvested. As pomegranate is a seasonal fruit, determining optimum storage conditions for fruit or its juice aiming at preserving its valuable nutritional components that may be reduced during storage, is important. In fact, during post harvesting storage, pomegranate displays important quality loss as a result of several physiological and enzymatic disorders with the major storage problem being water loss, leading to browning symptoms in both peel and arils [16, 49]. These symptoms increase at storage temperatures below 5 °C. Moreover, loss of turgidity, aril color, vitamin C and increased acidity have been reported, accompanied by a reduction in acceptability in terms of freshness, juiciness and taste [18].

Thermal processing (pasteurization) is the most used preservation technique to extend the shelf life of juices. However, this process may have adverse effects on sensory and nutritional values of juices therefore alternative preservation methods, which can destroy undesirable microorganisms with less adverse effects on product quality, urge. High Pressure Processing (HPP) is another technology shown to have several benefits such extension of food shelf-life, superior organoleptic quality and better nutrient retention to keep its original freshness, flavor, taste and color changes, which are minimal.

However, the results provided in this study show that when MDA cells were treated with PJ processed either with pasteurization or HPP technology the impact on the metabolic fluxes was completely different from that attained with fresh PJ. Indeed, we found a significant about 50% decrease of the mitochondrial oxidative metabolism with no significant effect on the glycolytic flux. So, even if it has been previously established that HPP treatments showed only a slight reduction in antioxidant capacity during storage time [29], this study unveils a loss of bioenergizing propriety of PJ undergoing these processes.

Storage under controlled atmosphere extends storage life of several agricultural products. It has been used successfully for maintaining quality of many tropical and sub-tropical fruit. Storage of fruit under controlled atmosphere and cold temperature reduces fruit respiration rate, ethylene production, suppresses or delays senescence processes and eventually increases postharvest shelf life [50]. When we evaluated the effect of PJ from pomegranate stored for 30 and 60 days in CA, we found a significant shift toward oxidative metabolism, more pronounced at 30 days, comparable with that attained with PJ extract from pomegranate immediately harvested. PJ extract from pomegranate stored in regular atmosphere RA showed a slight increase in oxidative metabolism without affecting glycolysis. Our results indicated that the best storage method enabling to mimic the bioenergizing properties of freshly extracted PJ is the one relying on conditions of CA for 30 days. Additionally, our data revealed that positive effect of PJ on mitochondrial respiration elicited by 30 days-storage under CA is independent on the temperature used, since enhancing the storage temperature from 4 to 8 °C show the same significant effect on the mitochondrial function.

However, the above reported effect of CA is completely lost after 60 days of storage with significant depression of both mitochondrial respiration and glycolysis. Intriguingly, and somehow surprising, storage under RA resulted even after 60 days in limited effects on the metabolic fluxes as compare with untreated cells.

To note PJ extract from packaged arils of pomegranate stored for 30 days in CA or RA, did not elicit the same positive effect on respiration, even if packaged arils stored at low temperatures retained appreciable nutritional and bioactive compound levels with low microbial count during the entire storage period [21, 51].

Freezing is another procedure used to preserve food products such as fruit juices. In the food industry, a storage temperature of − 18 °C effectively reduces chemical and microbial spoilage of foods. Nevertheless, frozen storage, even at − 25 °C, does not appear to preserve all the nutritional value of PJ. In fact evaluation of the total anthocyanin and phenolic contents in PJ stored at − 25 °C unveiled its decrease after 20 days’ frozen storage with consequent reduction of antioxidant activity [19]. However, when we analyzed the impact of frozen PJ on the ROS content of MDA cells we confirmed an antioxidant activity comparable with that observed following treatment with fresh PJ. Likewise, the proliferation rate of MDA-MB-231 cells was significantly dampened by frozen PJ treatment. To note, even relatively lower concentrations of frozen PJ were able to cause significant inhibition of cell growth as compared with the effect of fresh PJ and after a shorter incubation time.

However, treatment of MDA-MB-231 with frozen PJ was unable to recapitulate the metabolic shift observed with fresh PJ though conserving the capability to inhibit MDA proliferation. Treatment with frozen PJ resulted in inhibition of mitochondrial respiration and enhanced glycolysis at low and intermediate concentrations of PJ thereby causing also in this case a metabolic shift but promoting a more glycolytic/quiescent phenotype. Understanding the reasons for such conflicting results would require a “metabolomic” approach to assess the bioactive compounds present in the PJ before and after freezing to identify relevant changes in its composition. This was however beyond the aim of the presented study.

Based on what is reported in this study one is tempted to speculate that perhaps a balanced contribution of catabolic pathways such as glycolysis and mitochondrial oxidative routes is necessary beyond their bioenergetic outcome. Indeed, both glycolysis and tricarboxylic acid provide intermediates for biosynthesis of lipids, aminoacids, nucleotides as well as reducing equivalents. Therefore, it is possible that perturbing an established balance between different metabolic pathways might turn in dampening of the proliferative efficiency irrespective of the direction of the metabolic shift underwent (i.e. pro-glycolytic vs pro-oxidative).

## Conclusions

This study confirms the potential of pomegranate juice extract as a promising support in preventing cancer or even in assisting conventional onco-therapy as previously reported [9–11]. This appears mainly to rely on the herein confirmed powerful antioxidant properties of pomegranate chemicals. In addition, this study unveils the hiterto unappreciated effect of pomegranate juice constituents on the cellular metabolism and how post-harvested storage protocols differentially affect it.

However, at the same time, this study underlines the intrinsic difficulties in providing mechanistic insights when a multeplicity of effects are elicited by a mixture of bio-active compounds as those present in crude extracts. Untangling the specific contribution of the extract constituents or of their dosed mixture on selected biochemical cellular processes urges to improve the beneficial utilization of nature-provided compounds for preserving health.

### Supplementary Information

**Additional file 1: Fig. S1.** Effect of PJ from pomegranate stores in Controlled atmosphere (CA) on mitochondrial function in MDA cells. **Fig. S2.** Effect of PJ extracted from arils packaged for 5 days, previously extracted from pomegranates stored for 30 days in CA or RA, on mitochondrial function in MDA cells. **Fig. S3.** Effect of frozen PJ processed with thermal pasteurization (PT) and high hydrostatic pressure (HHP) on mitochondrial function in MDA cells. **Fig. S4.** Effect of frozen PJ from pomegranate stores at 4°C in Controlled atmosphere (CA) and in regular air (RA) on mitochondrial function in MDA cells. **Fig. S5.** Effect of frozen PJ from pomegranate stores at 8°C in Controlled atmosphere (CA) on mitochondrial function in MDA cells. **Fig. S6.** Effects of pH on cell viability, ROS production and metabolic fluxes in MDA cells.

## Data Availability

All data generated or analyzed during this study are included in this published article and its supplementary information fles are available from the corresponding authors on reasonable request.

## Abbreviations

CA-PJ: PJ extracted from controlled atmosphere –stored pomegranate whole fruit
DCF-DA: 2,7-Dichlorofluorescin diacetate
ECAR: Extracellular acidification rate
FCCP: Carbonyl cyanide 4-(trifluoromethoxy)phenylhydrazone
FZ-PJ: Frozen PJ
HPP-PJ: High pressure processed PJ
MDA: MDA-MB-231
NCI: Normalized cell index
OCR: Oxygen consumption rate
OXPHOS: Oxidative phosphorylation
PJ: Pomegranate juice
RA-PJ: PJ extracted from regular air -stored pomegranate whole fruit
ROS: Reactive oxygen species
TP-PJ: Thermal pasteurized PJ
