# Supplementary material for: Characterization of the effect of pomegranate crude extract, and its post-harvesting preservation procedures, on redox tone, cellular growth and metabolic profile of MDA-MB-231 cell line
Source: BMC Complement Med Ther. 2023 Sep 8;23:311. doi: 10.1186/s12906-023-04134-1 (PMC10485948; doi:10.1186/s12906-023-04134-1)

Supplementary figures

Fig. S1. Effect of PJ from pomegranate stores in Controlled atmosphere (CA) on mitochondrial function in MDA cells.

Cells were incubated 24 hours with PJ obtained from CA stored pomegranate fruit for 30 or 60 days at 8 °C and and analyzed in microplates by Seahorse. a) Statistical analysis of the OCRs obtained from Mitostress assay. The histograms show Basal, resting OCR; oligo, OCR in the presence of oligomycin; FCCP, OCR under uncoupled condition. Data, means ± SEM are expressed as percentage of control Basal OCR. b) The histograms show ATP turnover, maximal capacity and reserve capacity. Data, means ± SEM are expressed as percentage of control Basal OCR. c) Statistical analysis of glycolytic activity stress assay. The histograms show basal glycolysis, glycolytic reserve and glycolytic capacity. Data, means ± SEM are expressed as percentage of control Basal ECAR. d) Energy map was obtained plotting basal OCR versus glycolysis ECAR; * $p < 0.05$, ** $p < 0.01$; *** $p < 0.001$. Statistical significances are referred to control.

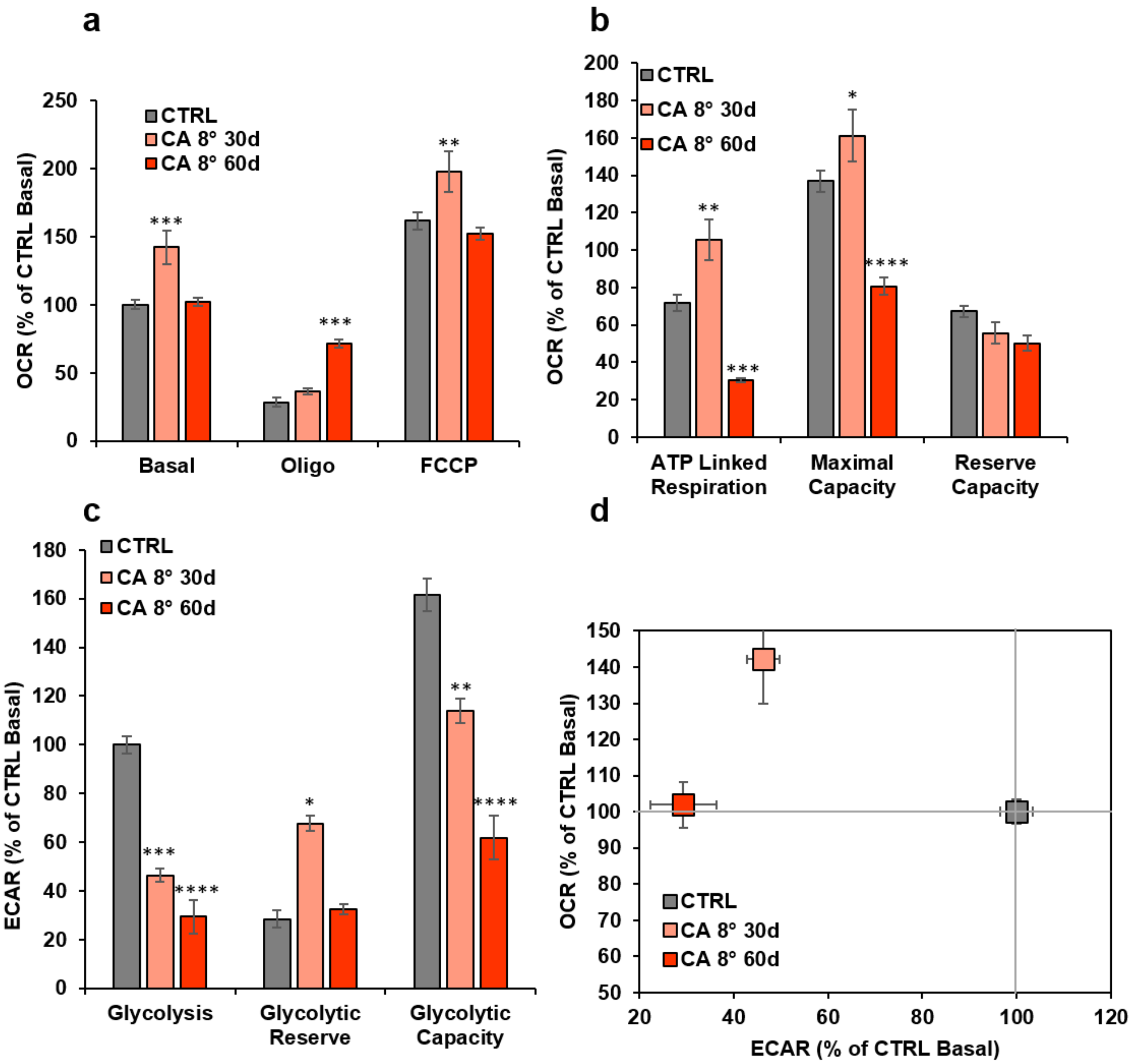

Fig. S2. Effect of PJ extracted from arils packaged for 5 days, previously extracted from pomegranates stored for 30 days in CA or RA, on mitochondrial function in MDA cells.

MDA were incubated 24 hours with 10% of PJ and analyzed in microplates by Seahorse. a) Statistical analysis of the OCRs obtained from Mitostress assay. The histograms show Basal, resting OCR; oligo, OCR in the presence of oligomycin; FCCP, OCR under uncoupled condition. Data, means ± SEM are expressed as percentage of control Basal OCR. b) The histograms show ATP turnover, maximal capacity and reserve capacity. Data, means ± SEM are expressed as percentage of control Basal OCR. c) Statistical analysis of glycolytic activity stress assay. The histograms show basal glycolysis, glycolytic reserve and glycolytic capacity. Data, means ± SEM are expressed as percentage of control Basal ECAR. d) Energy map was obtained plotting basal OCR versus glycolysis ECAR; * $p < 0.05$, ** $p < 0.01$; *** $p < 0.001$. Statistical significances are referred to control.

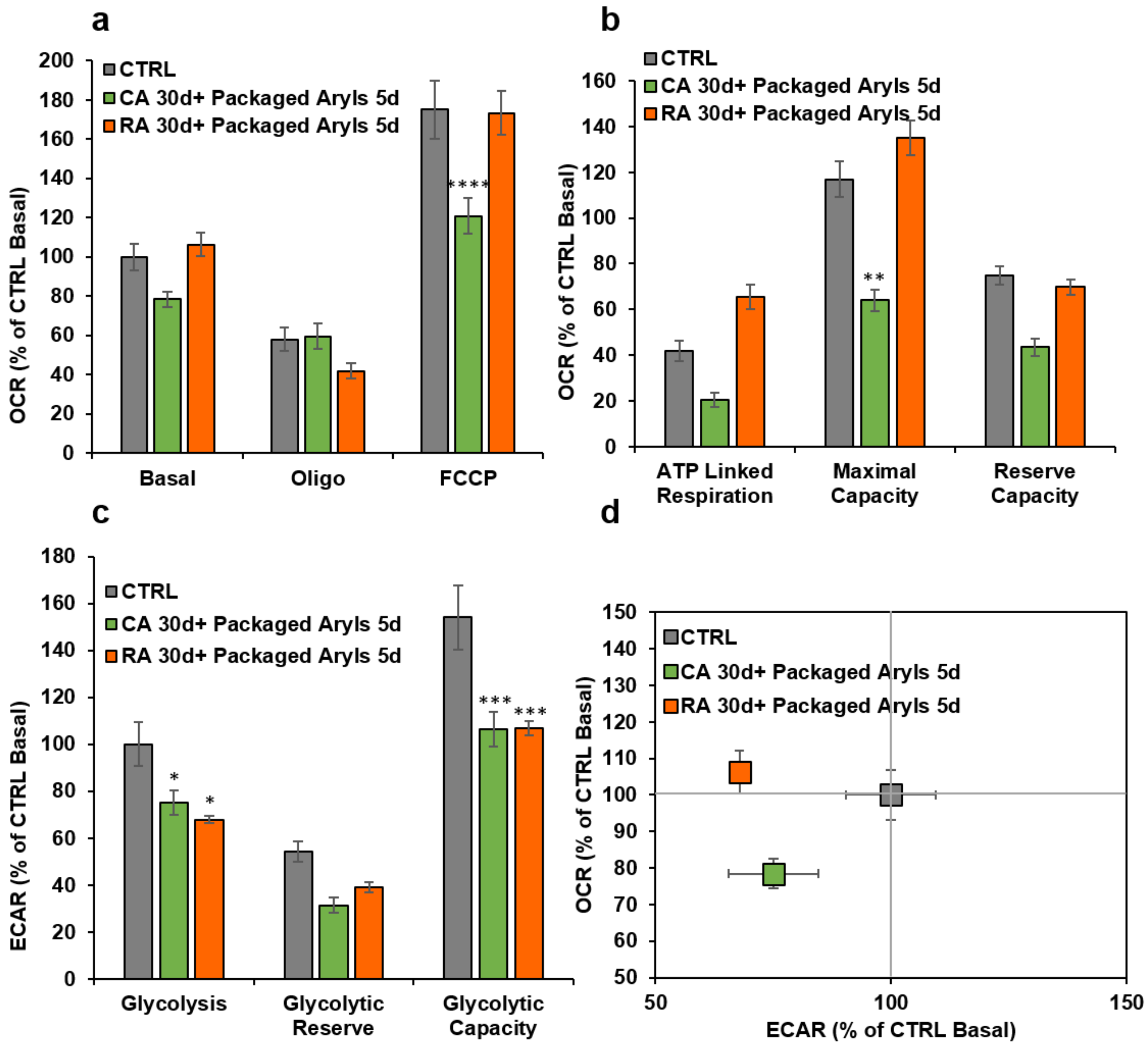

Fig. S3. Effect of frozen PJ processed with thermal pasteurization (PT) and high hydrostatic pressure (HHP) on mitochondrial function in MDA cells.

MDA were incubated 24 hours with frozen, thermal pasteurization (PT) or high pressure pasteurization (HPP) PJ and analyzed in microplates by Seahorse. a) Statistical analysis of the OCRs obtained from Mitostress assay. The histograms show Basal, resting OCR; oligo, OCR in the presence of oligomycin; FCCP, OCR under uncoupled condition. Data, means ± SEM are expressed as percentage of control Basal OCR. b) The histograms show ATP turnover, maximal capacity and reserve capacity. Data, means ± SEM are expressed as percentage of control Basal OCR (c) Statistical analysis of glycolytic activity stress assay. The histograms show basal glycolysis, glycolytic reserve and glycolytic capacity. Data, means ± SEM are expressed as percentage of control Basal ECAR. d) Energy map was obtained plotting basal OCR versus glycolysis ECAR; * $p < 0.05$, ** $p < 0.01$; *** $p < 0.001$. Statistical significances are referred to control.

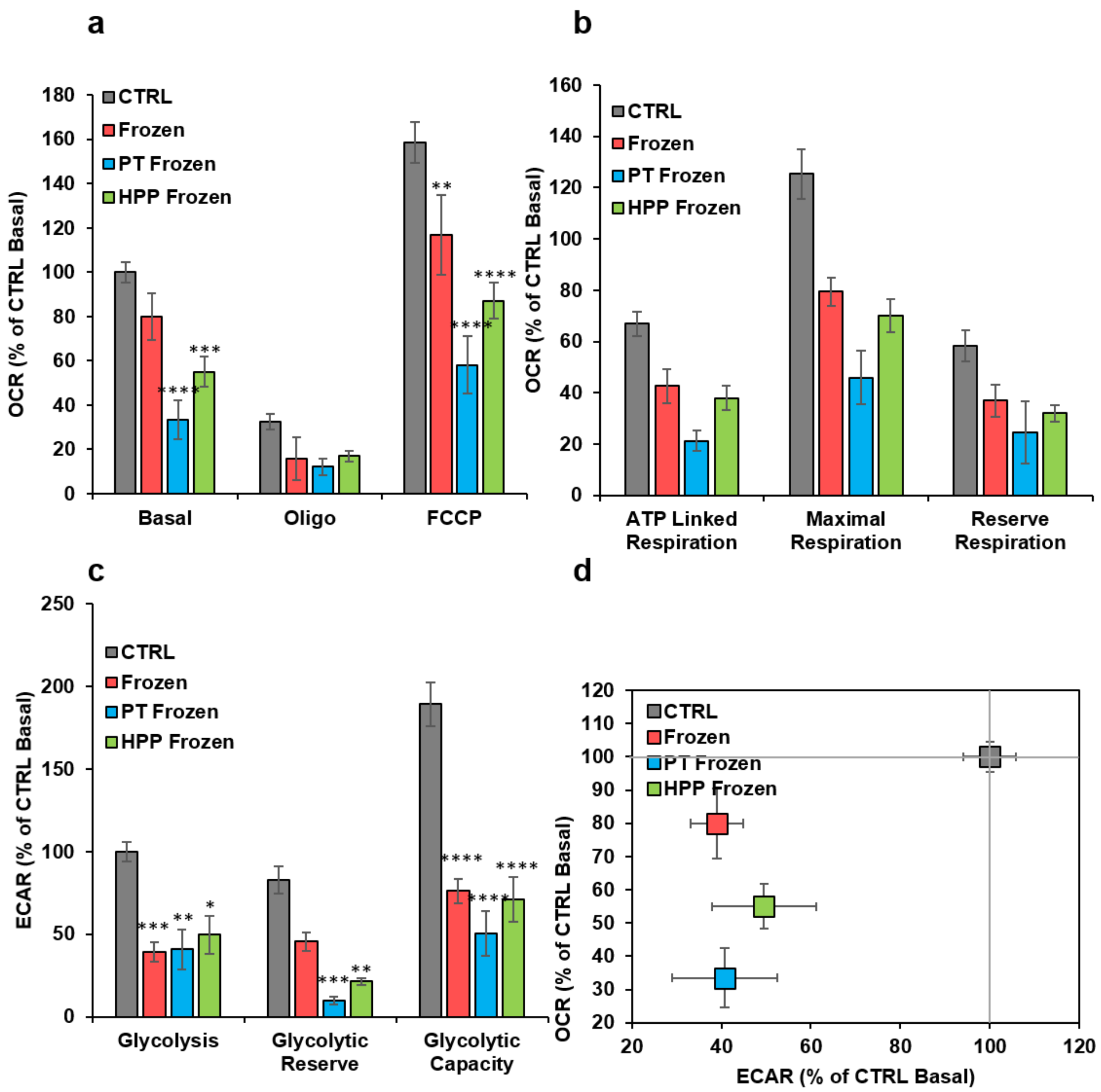

Fig. S4. Effect of frozen PJ from pomegranate stores at 4°C in Controlled atmosphere (CA) and in regular air (RA) on mitochondrial function in MDA cells.

Cells were incubated 24 hours with 30 days-frozen PJ extracted from pomegranates stored for 30 or 60 days in CA or for 30 days in RA and analyzed in microplates by Seahorse. a) Statistical analysis of the OCRs obtained from Mitostress assay. The histograms show Basal, resting OCR; oligo, OCR in the presence of oligomycin; FCCP, OCR under uncoupled condition. Data, means ± SEM are expressed as percentage of control Basal OCR. b) The histograms show ATP turnover, maximal capacity and reserve capacity. Data, means ± SEM are expressed as percentage of control Basal OCR. c) Statistical analysis of glycolytic activity stress assay. The histograms show basal glycolysis, glycolytic reserve and glycolytic capacity. Data, means ± SEM are expressed as percentage of control Basal ECAR. d) Energy map was obtained plotting basal OCR versus glycolysis ECAR; * $p < 0.05$, ** $p < 0.01$; *** $p < 0.001$. Statistical significances are referred to control.

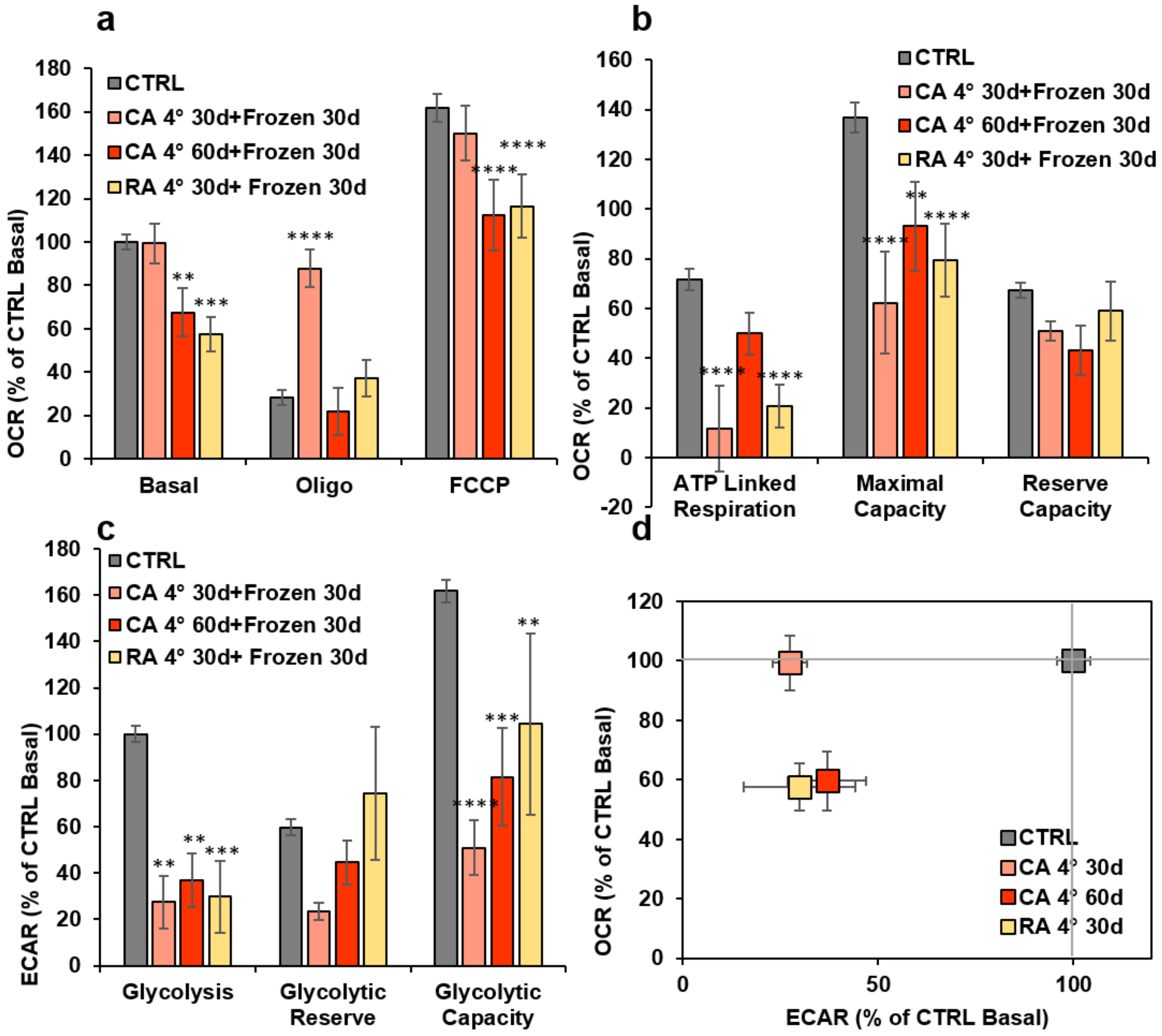

Fig. S5. Effect of frozen PJ from pomegranate stores at 8°C in Controlled atmosphere (CA) on mitochondrial function in MDA cells.

Cells were incubated 24 hours with 30 days-frozen PJ extracted from pomegranates stored for 30 or 60 days in CA 8°C and analyzed in microplates by Seahorse. a) Statistical analysis of the OCRs obtained from Mitostress assay. The histograms show Basal, resting OCR; oligo, OCR in the presence of oligomycin; FCCP, OCR under uncoupled condition. Data, means ± SEM are expressed as percentage of control Basal OCR. b) The histograms show ATP turnover, maximal capacity and reserve capacity. Data, means ± SEM are expressed as percentage of control Basal OCR. c) Statistical analysis of glycolytic activity stress assay. The histograms show basal glycolysis, glycolytic reserve and glycolytic capacity. Data, means ± SEM are expressed as percentage of control Basal ECAR. d) Energy map was obtained plotting basal OCR versus glycolysis ECAR; * $p < 0.05$, ** $p < 0.01$; *** $p < 0.001$. Statistical significances are referred to control.

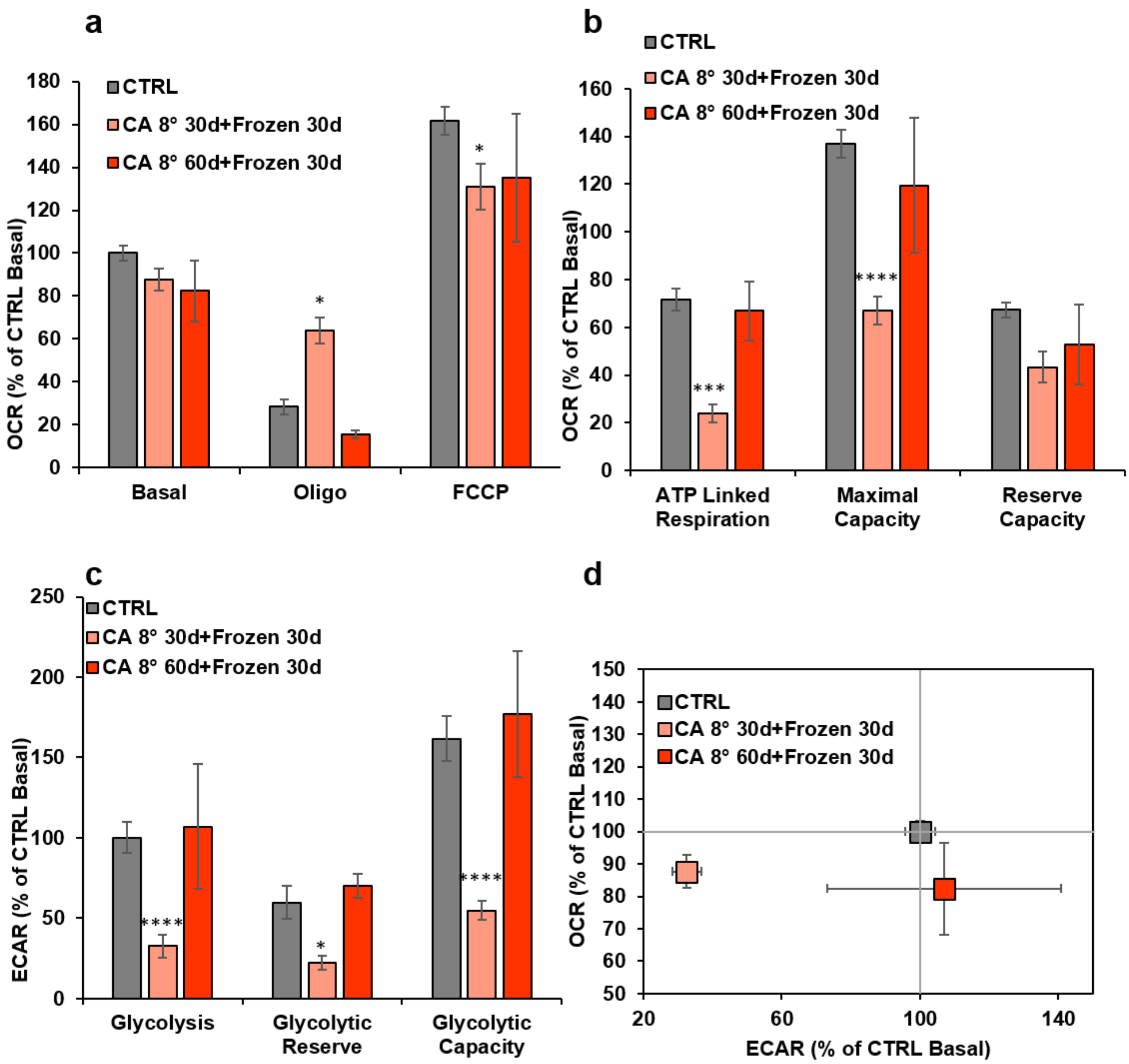

Fig. S6. Effects of pH on cell viability, ROS production and metabolic fluxes in MDA cells.

(a) Contrast phase imaging of MDA cells. Cells were grown in complete DMEM medium for approximately 24 h after seeding, then medium was changed, and cells were treated for 24 h with DMEM medium with regular pH 7.4 (CTRL), and DMEM with pH modified at the same values detected for 10% PJ (pH 7.0) (Acidified CTRL). (b) Laser scanning confocal microscopy (LSCM) imaging of DCF-probed ROS in MDA with the DMEMs described in (a). (c) Metabolic flux analysis. On the right the statistical analysis of mitochondrial respiration stress test assay is shown; the histogram displays basal OCR, OCR in the presence of oligomycin, OCR in the presence of FCCP in MDA incubated with regular ad acidified DMEM as described in (a) and following PJ treatment. On the left the statistical analysis of glycolytic activity stress assay is shown: the histogram displays basal glycolysis, glycolytic reserve and glycolytic capacity (see Material and Methods in the main text for further details). All data are represented as percentage versus control basal OCR or control glycolysis ECAR. The values are means ± SEM of four independent experiments. * $p < 0.05$; ** $p < 0.01$. Statistical significances are referred to control.

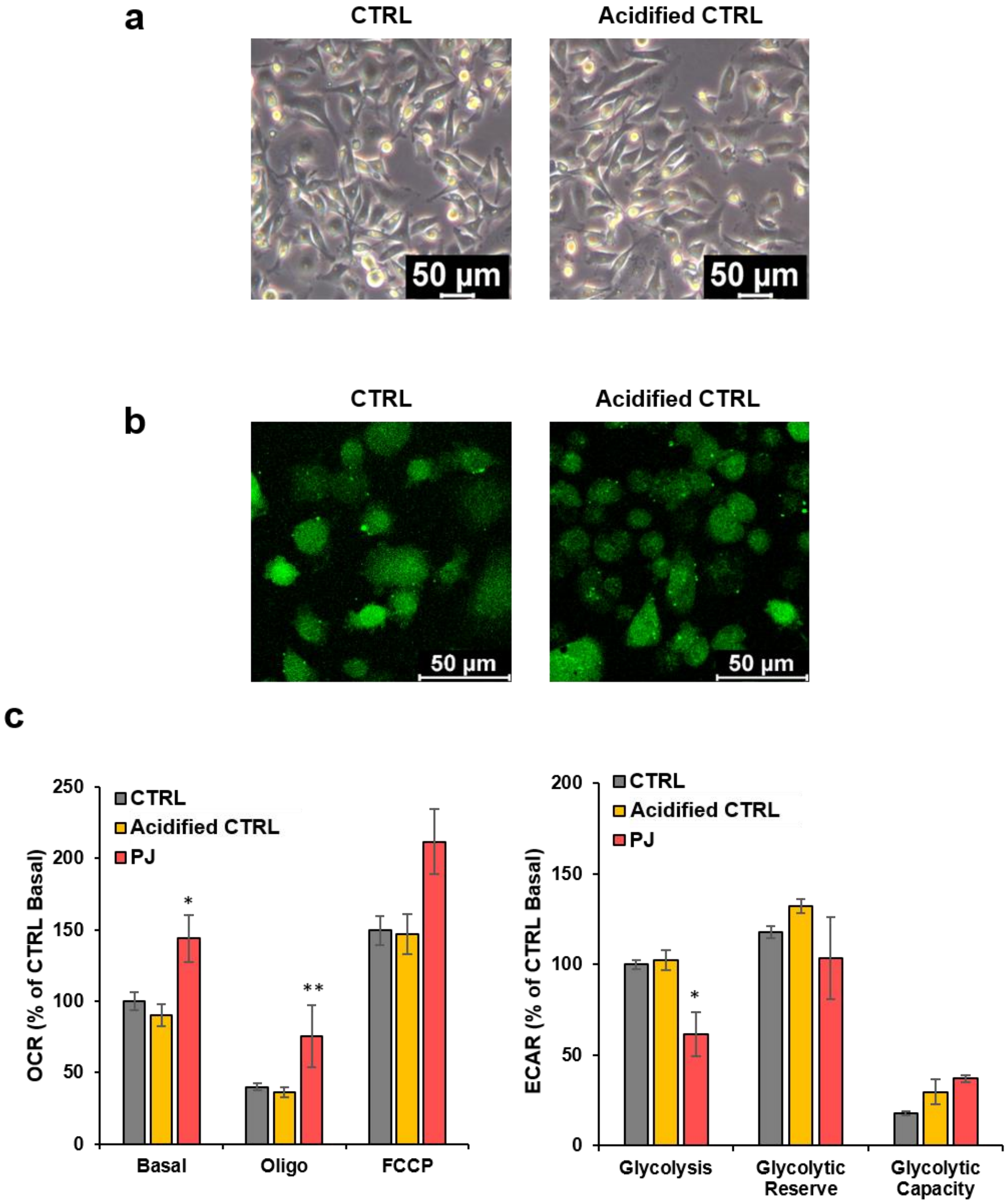

Supplement: Supplementary file 1 — Additional file 1: Fig. S1. Effect of PJ from pomegranate stores in Controlled atmosphere (CA) on mitochondrial function in MDA cells. Fig. S2. Effect of PJ extracted from arils packaged for 5 days, previously extracted from pomegranates stored for 30 days in CA or RA, on mitochondrial function in MDA cells. Fig. S3. Effect of frozen PJ processed with thermal pasteurization (PT) and high hydrostatic pressure (HHP) on mitochondrial function in MDA cells. Fig. S4. Effect of frozen PJ from pomegranate stores at 4°C in Controlled atmosphere (CA) and in regular air (RA) on mitochondrial function in MDA cells. Fig. S5. Effect of frozen PJ from pomegranate stores at 8°C in Controlled atmosphere (CA) on mitochondrial function in MDA cells. Fig. S6. Effects of pH on cell viability, ROS production and metabolic fluxes in MDA cells. [file 12906_2023_4134_MOESM1_ESM.pdf]
